# Anesthesia Considerations for a Patient on Semaglutide and Delayed Gastric Emptying

**DOI:** 10.7759/cureus.42153

**Published:** 2023-07-19

**Authors:** Erina Fujino, Kathryn W Cobb, Jay Schoenherr, Lindsey Gouker, Elisa Lund

**Affiliations:** 1 Anesthesiology, University of North Carolina at Chapel Hill School of Medicine, Chapel Hill, USA

**Keywords:** pulmonary aspiration, anesthesia, delayed gastric emptying, glp-1, glucagon-like peptide-1 receptor agonist, semaglutide

## Abstract

Semaglutide is a class of long-acting glucagon-like peptide-1 receptor agonists (GLP1-RA) used for the treatment of type 2 diabetes mellitus (T2DM) and obesity. We present a 31-year-old female patient with a past medical history of T2DM without complication and no long-term or current use of insulin, class 3 obesity, hypertension, hyperlipidemia, polycystic ovary syndrome (PCOS), and anxiety, who underwent an esophagogastroduodenoscopy (EGD) in preparation for bariatric surgery while taking semaglutide. Despite appropriately following the preoperative fasting guidelines of the American Society of Anesthesiologists (ASA), endoscopy revealed food residue in the gastric body, necessitating abortion of the procedure to reduce the risk of intraoperative pulmonary aspiration. Given the lack of preoperative fasting guidelines for patients on semaglutide to date, and delayed gastric emptying being a known side effect among patients taking semaglutide, anesthesiologists should be aware of alternative methods to ensure no food is present in the stomach to mitigate the risk of pulmonary aspiration during general anesthesia.

## Introduction

Semaglutide is a class of long-acting glucagon-like peptide-1 receptor agonists (GLP1-RA) used in the management of type 2 diabetes mellitus (T2DM) and obesity. Ozempic® (semaglutide, Novo Nordisk Inc., Plainsboro, USA), which was approved by the Food and Drug Administration (FDA) in 2017 for the treatment of T2DM, garnered widespread attention after social media influencers described significant weight loss when used off-label for this purpose. In 2021, the FDA approved semaglutide under the brand name Wegovy® (Novo Nordisk Inc., Plainsboro, USA), for weight management in patients with obesity or overweight with at least one weight-related condition, such as high blood pressure, T2DM, or high cholesterol. Future semaglutide demand is expected to increase due to the rise in adolescent obesity and its association with diagnoses of T2DM [1-2]. 

Semaglutide selectively binds to and activates glucagon-like peptide-1 receptors, increasing insulin and suppressing glucagon secretion and requiring only once-weekly injection [3]. Recent studies also report that patients on semaglutide reduced their caloric intake and lost weight by delaying gastric emptying, although this practice is controversial [4-7]. The effects of delayed gastric emptying are particularly concerning for patients scheduled to undergo procedures requiring adherence to specific fasting guidelines prior to anesthesia. Current practice guidelines from the American Society of Anesthesiologists (ASA) state that for healthy adults, one should consider a minimum fasting duration of two hours for clear fluids, six hours for a light meal, and eight hours for a fatty meal, fried foods, or meat to safely conduct anesthesia [8]. In the clinical setting, however, anecdotally, we have noted an increased incidence of gastric food retention among patients on semaglutide despite appropriately following preoperative fasting recommendations. To date, few case reports have evaluated the safety of semaglutide with respect to the risk of unknown retained gastric contents among patients presenting for anesthesia [9-10]. We present a patient on semaglutide who followed the standard recommended preoperative fasting protocol but intraoperatively was discovered to have retained gastric contents necessitating abortion of her procedure.

## Case presentation

A 31-year-old female with a past medical history of T2DM without complication and no long-term current use of insulin (hemoglobin A1c 5.9%), class 3 obesity (136 kg, BMI 45.6), hypertension, hyperlipidemia, polycystic ovary syndrome (PCOS), and anxiety, presented to a Weight Management Clinic. She was previously on metformin but discontinued this medication over three months ago. She had also elected to stop taking metoprolol and medroxyprogesterone (Provera, AbbVie Inc., North Chicago, USA), for hypertension and PCOS, respectively. She had no history of gastroesophageal reflux disease or gastric surgery. She expressed interest in bariatric surgery to improve her health and was referred to the Bariatric Surgery Clinic. As part of her weight loss regimen, she began weekly injections of 0.25 milligrams (mg) of semaglutide (Ozempic) and was started on medical nutrition therapy.  

**Figure 1:**
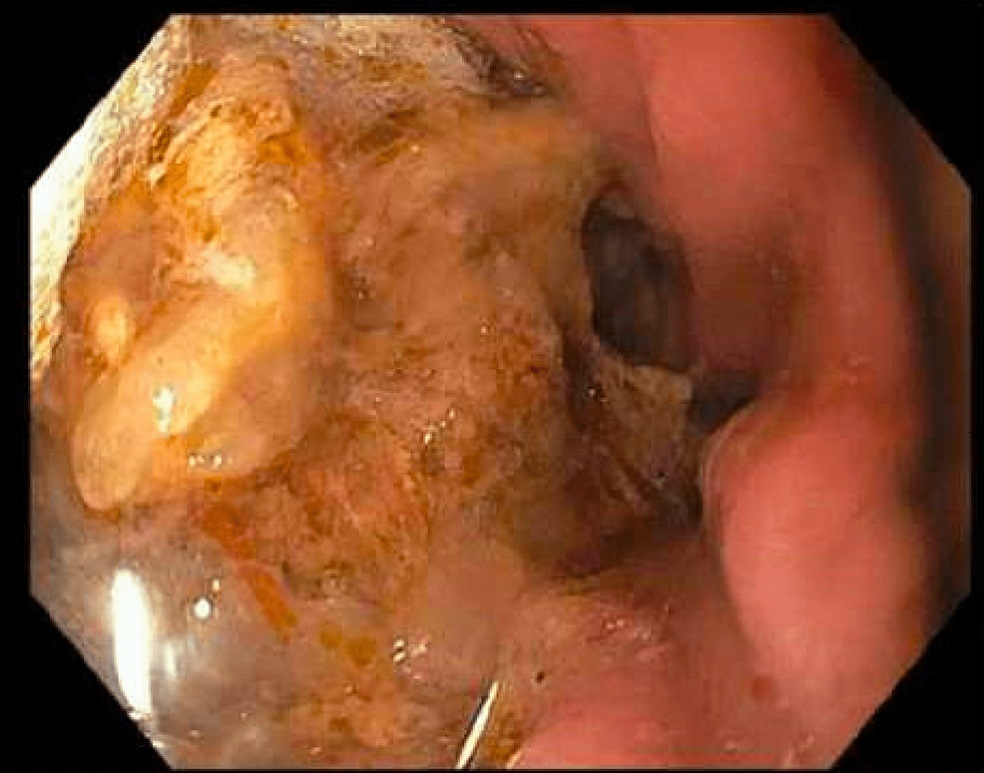
EGD showing the presence of food in the stomach despite fasting to solids for over 10 hours. EGD: esophagogastroduodenoscopy

One month after the initial consultation, the patient presented for an esophagogastroduodenoscopy (EGD) as part of her preoperative assessment. She had been instructed to eat nothing after midnight and by the time of the procedure, she had been fasting to solid food for over 10 hours. The patient continued to use Ozempic as prescribed prior to the EGD and did not complain of any new gastric symptoms. Prior to induction of anesthesia, standard monitors were applied, and the patient was placed in the left lateral, semi-recumbent position with the plan for a native airway general anesthetic with propofol infusion. Oxygen was delivered via a procedural face mask and a bite block as placed. Anesthesia was induced with 40 mg of lidocaine and 150 mg of propofol and a propofol infusion of 200 mcg/kg/min was started. One minute later, the procedure was started. When the endoscope reached the stomach, a large amount of food was found in the gastric body (Figure 1). The procedure was aborted given the absence of a secure airway and the high risk of aspiration. The suction was readily available in the case of aspiration. The propofol infusion was stopped and the patient emerged from anesthesia uneventfully. 

**Figure 2:**
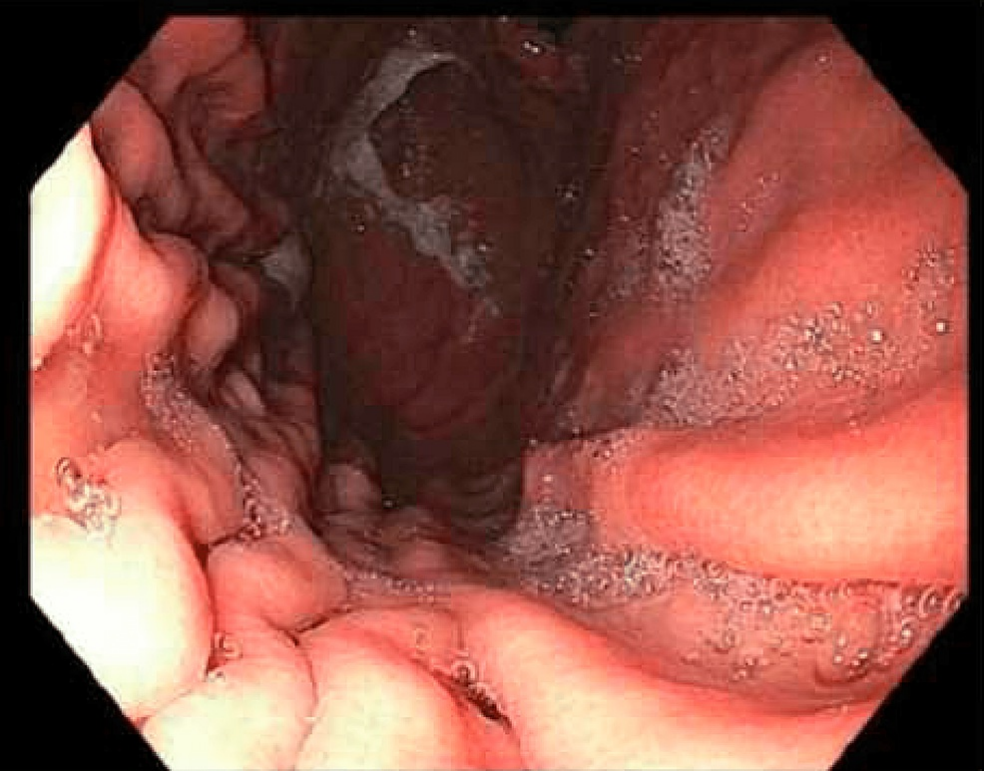
EGD showing no presence of food after adhering to a liquid diet for 36 hours before the repeat procedure. EGD: esophagogastroduodenoscopy

One month after the aborted EGD procedure, the patient was instructed to adhere to a liquid diet for 36 hours before the repeat EGD. Her last dose of semaglutide was also reported to be seven days prior to the procedure day. The patient was compliant with the new fasting instructions, and the same anesthetic plan was conducted as in the first EGD. No food was present in the stomach during the procedure (Figure 2).

## Discussion

Semaglutide has increased in popularity and accessibility in recent years, resulting in patients more frequently presenting for surgery while taking this medication. In this case report, we discuss a T2DM patient without diagnoses of gastroparesis nor any gastric symptoms on semaglutide with delayed gastric emptying during her elective EGD, requiring additional precautions to minimize the risk of an aspiration event.

There are currently seven approved GLP1-RA medications, which work by different mechanisms of action based upon their duration of action (long or short). The short-acting agents, including exenatide and lixisenatide, primarily work by delaying gastric emptying to suppress post-prandial hyperglycemia. Long-acting agents, including liraglutide, exenatide long-acting release, dulaglutide, and semaglutide increase insulin secretion and suppress glucagon to manage postprandial hyperglycemia. The effects of gastric emptying also vary between these two classes, as short-acting GLP1-RA decelerate gastric emptying compared to long-acting GLP1-RA [11-12]. However, clinically, a recent study suggests that patients on long-acting GLP1-RA have a significantly higher proportion of gastric residue in comparison to the non-GLP1-RA treatment group measured by EGD in patients with diabetes, indicating that the effect on gastric emptying even for a patient on a long-acting GLP1-RA may be significant [6].

In our patient, we speculate that semaglutide therapy initiation may be one of the predisposing factors associated with the presence of gastric residue. While it is possible that our patient may have had diabetic gastroparesis at baseline as both type 1 and type 2 diabetes are frequently linked to abnormal delayed gastric emptying, it is worth noting that the patient did not have a diagnosis of gastroparesis nor have reported any symptoms frequently experienced by patients with diabetic gastroparesis, such as postprandial dyspepsia, early satiety, nausea, vomiting, heartburn, bloating, and pain [13-14]. Additionally, in a well-controlled, uncomplicated T2DM, gastric emptying can be accelerated rather than delayed [15]. Furthermore, individuals with morbid obesity can have accelerated gastric emptying to solid food compared to individuals with a healthy weight [16]. More recently, semaglutide is investigated to see whether there is a dose-dependent medication-induced delayed gastric emptying, especially at high doses (≥1.0 mg) [3,7]. While our patient received an introductory dose of 0.25 mg of semaglutide, given the trends we have been seeing with the use of semaglutide, it is worth considering that even at low doses, semaglutide could have delayed gastric emptying more than simply exacerbating diabetic gastroparesis.

This case highlights the potential need for additional aspiration precautions for patients taking semaglutide, as retention of solid gastric contents is one of the biggest predisposing risk factors for aspiration. Although pulmonary aspiration is a rare intraoperative event, it can lead to significant morbidity and mortality. In addition, patients undergoing general anesthesia with common anesthetic agents, such as propofol and opioids, have an increased risk of aspiration due to the loss of protective airway reflex from the depression of the lower esophageal sphincter tone [17]. At this point, there is no significant clinical evidence to necessitate changes in preoperative fasting guidelines or management. Nevertheless, clinicians may use their judgment to promote the safety of a patient on semaglutide, such as placing the patient on a liquid diet for 36 hours prior to EGD or adding prokinetic drugs, such as dopamine antagonist metoclopramide or motilin agonist erythromycin. Perhaps, patients could also stop taking the drug prior to the surgery; however, given that the half-life of semaglutide is approximately seven days, patients would need to be off the drug for more than five weeks, which could raise concerns for poor glycemic control and increased cardiovascular risks [11,18].

Going forward, prevention measures such as more conservative fasting recommendations and additional preoperative evaluation of gastric contents using ultrasound could be employed [19]. As such, future research is needed to assess whether this case represents a true trend of increased risk to delayed gastric emptying in the preoperative setting or merely a correlation with diabetic gastroparesis for patients taking semaglutide. Examples include a retrospective study determining whether different doses of semaglutide have an effect on the likelihood of retained gastric content in patients diagnosed with type 1 or type 2 diabetes, or a prospective study utilizing ultrasound to examine gastric contents in diabetic patients on semaglutide prior to general anesthesia.

## Conclusions

Due to an increased frequency of prescription of semaglutide in recent years, anesthesiologists are more often caring for patients taking semaglutide. This case report highlights delayed gastric emptying accentuated in an obese, well-controlled T2DM patient taking semaglutide, posing an increased risk of gastric aspiration under general anesthesia. While regular fasting guidelines may not be adequate to prevent the risk of perioperative aspiration, implementing extended fasting period, prescribing prokinetic medications, utilizing preoperative ultrasound assessment of gastric content, and halting the use of semaglutide prior to the procedure may mitigate the risk of perioperative pulmonary aspiration.
